# Acute Postoperative Pain Control

**DOI:** 10.1155/2017/7831014

**Published:** 2017-06-08

**Authors:** Volkan Hancı, Bülent S. Yurtlu, Rudin Domi, Yasuyuki Shibata, Can Eyigör

**Affiliations:** ^1^Department of Anesthesiology and Reanimation, Dokuz Eylül University, İzmir, Turkey; ^2^Department of Anesthesiology and Reanimation, Mother Teresa University Hospital Center, Tirana, Albania; ^3^Department of Anesthesiology and Reanimation, Nagoya University Hospital, Nagoya, Japan; ^4^Department of Anesthesiology and Reanimation, Ege University, İzmir, Turkey

Recovery from anesthesia is a source of great stress for patients. Postanesthesia recovery must occur comfortably and uneventfully in a controlled environment. However, as it usually starts in the operating room, patients are brought to the recovery unit with airway blockage, shiver, agitation, delirium, pain, nausea, vomiting, hypothermia, and autonomic instability risks. Most surgical morbidities and mortalities happen in the postoperative period. Among patients who have had surgery, the early and late postoperative periods constitute one of the most critical stages. All of these facts emphasize the importance of postoperative recovery and postoperative care [1]. Millions of surgical therapies are performed worldwide and patients suffer from varying degrees of postoperative pain after these procedures [2]. Postoperative pain is one of the most important problems in the postoperative period. Postoperative pain, with nociceptive, inflammatory, and neuropathic components, begins with surgical trauma and reduces as the tissue heals. Untreated pain caused by surgical trauma produces very important physiopathologic changes in children and adults. Ešective treatment of postoperative pain decreases surgical mortality and morbidity rates and has been shown to promote quicker healing [2]. One of the main components of early recovery after surgery programs is establishment of adequate perioperative analgesia. Historically, systemic administration of opioid drug groups has been the cornerstone of acute postoperative pain control but trends have changed towards increased utilization of regional anesthetic and analgesic techniques during the last few decades. Of note, in the last decade, even the regional techniques affecting a big surface area of the body have been questioned together with the drugs or drug combinations used for them. There is an instance of looking for new or modified techniques or drug combinations in hope of reduced side effect but increased efficiency [2–6]. In this specific issue, the readers will find eleven articles opening the gate bigger to our understanding of acute pain management after surgery. These articles cover some of the missing parts on postoperative pain management subject.

In this issue, readers will find eleven articles including “The Effect of Peritubal Infiltration with Bupivacaine and Morphine on Postoperative Analgesia in Patients Undergoing Percutaneous Nephrolithotomy” by I. Karaduman and colleagues, “The Antinociceptive Effect of Light-Emitting Diode Irradiation on Incised Wounds Is Correlated with Changes in Cyclooxygenase 2 Activity, Prostaglandin E2, and Proinflammatory Cytokines” by Y.-Y. Chia and colleagues, “Gabapentin Does Not Appear to Improve Postoperative Pain and Sleep Patterns in Patients Who Concomitantly Receive Regional Anesthesia for Lower Extremity Orthopedic Surgery: A Randomized Control Trial” by J. D. Eloy and colleagues, “Peritoneal Nebulization of Ropivacaine during Laparoscopic Cholecystectomy: Dose Finding and Pharmacokinetic Study” by M. Allegri and colleagues, “Adherence to All Steps of a Pain Management Protocol in Intensive Care Patients after Cardiac Surgery Is Hard to Achieve” by L. Gulik and colleagues, “The Anatomic Relationship of the Tibial Nerve to the Common Peroneal Nerve in the Popliteal Fossa: Implications for Selective Tibial Nerve Block in Total Knee Arthroplasty” by E. R. Silverman and colleagues, “Comparison of the Effects of Intrathecal Fentanyl and Intrathecal Morphine on Pain in Elective Total Knee Replacement Surgery” by R. Kılıçkaya and colleagues, “Combination Therapy with Continuous Three-in-One Femoral Nerve Block and Periarticular Multimodal Drug Infiltration after Total Hip Arthroplasty” by T. Tetsunaga and colleagues, “Postthoracotomy Ipsilateral Shoulder Pain: A Literature Review on Characteristics and Treatment” by F. Yousefshahi and colleagues, “Effect of Intravenous High Dose Vitamin C on Postoperative Pain and Morphine Use after Laparoscopic Colectomy: A Randomized Controlled Trial” by Y. Jeon and colleagues, and “A Comparison of Oxycodone and Alfentanil in Intravenous Patient-Controlled Analgesia with a Time-Scheduled Decremental Infusion after Laparoscopic Cholecystectomy” by Y. S. Kwon and colleagues.

This body of research as a whole emphasizes the importance of acute pain management after surgery and fills out the knowledge gaps in specific areas and presents new alternate therapies when applicable.

*Volkan Hancı* *Volkan Hancı* *Bülent S. Yurtlu* *Bülent S. Yurtlu* *Rudin Domi* *Rudin Domi* *Yasuyuki Shibata* *Yasuyuki Shibata* *Can Eyigör* *Can Eyigör*
